# Tetra­ethyl­ammonium tetra­kis­(1,1,1,5,5,5-hexa­fluoro­acetyl­acetonato)terbate(III)

**DOI:** 10.1107/S1600536811055437

**Published:** 2012-01-07

**Authors:** Rik Van Deun, Pascal Van Der Voort, Isabel Van Driessche, Kristof Van Hecke

**Affiliations:** a Department of Inorganic and Physical Chemistry, Ghent University, Krijgslaan 281–S3, B-9000 Ghent, Belgium

## Abstract

The title compound, (C_8_H_20_N)[Tb(C_5_HF_6_O_2_)_4_], is a tetrakis β-diketonate complex of hexa­fluoro­acetyl­acetone with terbium(III), and tetra­ethyl­ammonium as the counter-ion. This compound shows typical green terbium(III) luminescence upon excitation at about 335 nm. The coordination geometry around the Tb^III^ atom is a slightly distorted square anti­prism. One hexa­fluoro­acetyl­acetone ligand has a disordered CF_3_ group [occupancies of 0.575 (4) and 0.425 (4)]. A three-dimensional network is built up by linkage of Tb^III^ complexes *via* C—H⋯F inter­actions.

## Related literature

For a review on rare-earth β-diketonate complexes, their crystal structures and applications, see: Binnemans (2005 ▶). We have widely studied rare-earth β-diketonate complexes for their luminescence properties (Mech *et al.*, 2008 ▶; Van Deun *et al.*, 2007 ▶), either as pure materials, doped in liquid crystals (Van Deun *et al.*, 2003 ▶; Nockemann *et al.*, 2005 ▶), or processed into thin films (Lenaerts *et al.*, 2005 ▶, O’Riordan *et al.*, 2005 ▶). For related structures, see: Tang & Mudring (2009 ▶); Danford *et al.* (1970 ▶); Lunstroot *et al.* (2009 ▶); Mehdi *et al.* (2010 ▶). For general procedues for the synthesis of rare-earth β-diketonate complexes, see: Melby *et al.* (1964 ▶). For a description of the Cambridge Structural Database, see: Allen (2002 ▶).
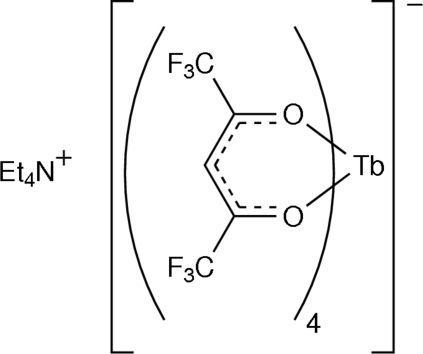


## Experimental

### 

#### Crystal data

(C_8_H_20_N)[Tb(C_5_HF_6_O_2_)_4_]
*M* *_r_* = 1117.41
Monoclinic, 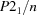

*a* = 12.7113 (9) Å
*b* = 16.9355 (13) Å
*c* = 18.3540 (11) Å
β = 94.657 (6)°
*V* = 3938.1 (5) Å^3^
*Z* = 4
Mo *K*α radiation
μ = 1.96 mm^−1^
*T* = 100 K
0.4 × 0.1 × 0.1 mm

#### Data collection

Agilent SuperNova Dual Cu at zero Atlas diffractometer
Absorption correction: multi-scan (*CrysAlis PRO*; Agilent, 2010 ▶) *T*_min_ = 0.531, *T*_max_ = 0.820
14178 measured reflections
6876 independent reflections
4772 reflections with *I* > 2σ(*I*)
*R*_int_ = 0.064

#### Refinement

*R*[*F* ^2^ > 2σ(*F* ^2^)] = 0.054
*wR*(*F* ^2^) = 0.123
*S* = 0.98
6876 reflections
591 parameters
90 restraints
H-atom parameters constrained
Δρ_max_ = 1.51 e Å^−3^
Δρ_min_ = −1.24 e Å^−3^

### 

Data collection: *CrysAlis PRO* (Agilent, 2010 ▶); cell refinement: *CrysAlis PRO*; data reduction: *CrysAlis PRO*; program(s) used to solve structure: *SHELXS97* (Sheldrick, 2008 ▶); program(s) used to refine structure: *SHELXL97* (Sheldrick, 2008 ▶); molecular graphics: *DIAMOND* (Brandenburg, 2008 ▶); software used to prepare material for publication: *PLATON* (Spek, 2009 ▶).

## Supplementary Material

Crystal structure: contains datablock(s) I, global. DOI: 10.1107/S1600536811055437/pk2378sup1.cif

Structure factors: contains datablock(s) I. DOI: 10.1107/S1600536811055437/pk2378Isup2.hkl

Additional supplementary materials:  crystallographic information; 3D view; checkCIF report

## Figures and Tables

**Table 1:** Selected bond lengths (Å)

Tb1—O1	2.373 (3)
Tb1—O2	2.351 (4)
Tb1—O3	2.345 (3)
Tb1—O4	2.369 (4)
Tb1—O5	2.372 (3)
Tb1—O6	2.351 (3)
Tb1—O7	2.359 (4)
Tb1—O8	2.365 (3)

**Table 2:** Hydrogen-bond geometry (Å, °)

*D*—H⋯*A*	*D*—H	H⋯*A*	*D*⋯*A*	*D*—H⋯*A*
C13—H13⋯F20^i^	0.95	2.51	3.430 (6)	164
C21—H21*A*⋯F10^ii^	0.99	2.47	3.279 (7)	139
C26—H26*C*⋯F14^iii^	0.98	2.49	3.451 (7)	169
C27—H27*B*⋯F2*A*^iii^	0.99	2.48	3.371 (9)	149

Symmetry codes: (i) 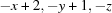; (ii) 
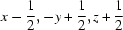
; (iii) 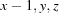.

**Fig. 1.**
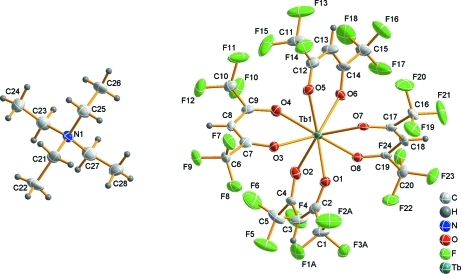
Coordination geometry of the title compound, showing 50% probability displacement ellipsoids. The disorder of one of the CF3 groups is not shown.

**Fig. 2.**
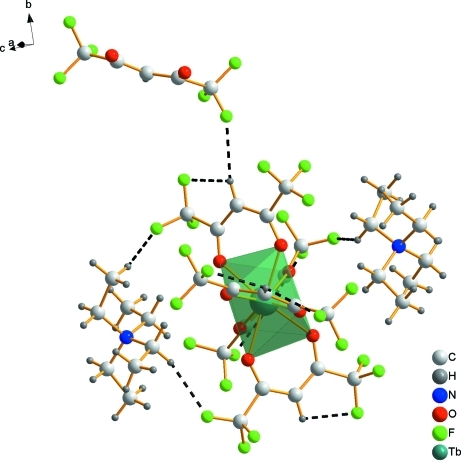
Intraanion, interanion-cation and interanion-anion C–H···F interactions (dashed lines), observed in the crystal structure of the title compound.

**Fig. 3.**
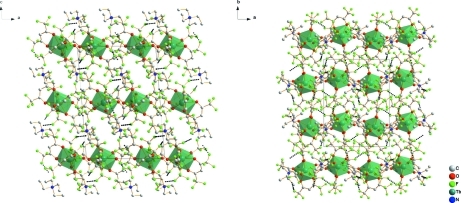
Packing diagram of the title compound along the b-axis, showing the two-dimensional layer formed in the (010)-plane (left) and along the c-axis, showing the three-dimensional network, with the hfac CF3 groups at the interfaces of the layers (right). The interanion-cation and interanion-anion C–H···F interactions are indicated. H-atoms were omitted to enhance clarity.

## supplementary crystallographic information

### Comment

β-diketones (1,3-diketones) are able to coordinate, as conjugate bases, to rare-earth ions, forming the corresponding β-diketonate complexes. Because of the accessibility to different commercially available β-diketones and the fact that the derived rare-earth complexes are relatively easy to synthesize, these β-diketonates have become the most scientifically studied and the most popular rare-earth coordination compounds.

For instance, rare-earth β-diketonates have been investigated as extractants in solvent-solvent extraction processes, as NMR shift reagents, as active materials in liquid lasers and novel types of organic light-emitting diodes (OLEDs), as active compounds in electroluminescent devices (*e.g.* flat-panel displays), as luminescent probes in bioassays, as precursors for chemical vapor deposition and as catalysts in organic reactions. These rare-earth β-diketonate complexes can be grouped into three main types: tris complexes, Lewis base adducts of the tris complexes (or ternary rare-earth β-diketonates) and tetrakis complexes.

An overiew of the different types of rare-earth β-diketonate complexes, their crystal structures and applications, is given by Binnemans, 2005.

We have widely studied rare-earth β-diketonate complexes for their luminescence properties (Mech *et al.*, 2008; Van Deun *et al.*, 2007), either as pure materials, doped in liquid crystals (Van Deun *et al.*, 2003; Nockemann *et al.*, 2005), or processed into thin films (Lenaerts *et al.*, 2005, O'Riordan *et al.*, 2005) and have recently determined other tetrakis rare-earth β-diketonate complexes with hexafluoroacetylacetone ligands (Lunstroot *et al.*, 2009; Mehdi *et al.*, 2010).

Here, we describe the crystal structure of a tetrakis complex of hexafluoroacetylacetone (hfac) with the terbium cation, Tb(III), and tetraethylammonium (Et_4_N) as the counter ion, which shows typical green Tb(III) luminescence upon excitation at about 335 nm.

The title compound crystallizes in the monoclinic space group *P*2_1_/n, with four formula units in the unit cell. The asymmetric unit consists of one Tb(III) cation, four hfac anions and one Et_4_N cation, which in total equals one formula unit. Each Tb(III) ion is eight-coordinated by oxygen atoms from four chelating hfac ligands. The coordination polyhedron around Tb(III) can be best described as a slightly distorted square antiprism (Figure 1). There are no solvent molecules coordinating to the Tb(III) ion. One of the CF_3_ groups of one of the hfac ligands is found disordered. The Tb–O distances range from 2.345 (3) to 2.373 (3) Å, which are comparable to those reported for other tetrakis(acetylacetonato)-Tb(III) complexes (Tang & Mudring, 2009) (Table 1). The O–Tb–O angles range from 73.81 (12)° to 75.19 (12)°. The only other tetrakis(acetylacetonato)-Tb(III) complex, found in the Cambridge Structural Database (CSD) has a Cs^+^ counterion (Danford *et al.*, 1970). However, no coordinates are available for the latter structure (reference code QQQBZM, CSD (Version 5.32) (Allen, 2002)).

No classic hydrogen bonds are found. However, C–H···F potential hydrogen bonds can be observed within the [Tb(hfac)_4_]^-^ anion itself (intraanion), between the [Tb(hfac)_4_]^-^ anion and [Et_4_N]^+^ cations (interanion-cation), as well as between different [Tb(hfac)_4_]^-^ anions (interanion-anion). The acidic hydrogen atom in each hfac ligand forms at least one intraanion hydrogen bond with a fluorine atom of one of its adjacent CF_3_ groups (C(–H)···F distances ranging from 2.713 (7) to 2.743 (6) Å). Several interanion-cation hydrogen bonds are observed between the [Tb(hfac)_4_]^-^ anion and the [Et_4_N]^+^ cations ((C(–H)···F distances ranging from 3.279 (7) to 3.451 (7) Å). Furthermore, one acidic hfac proton forms a C–H···F intermolecular interanion-anion hydrogen bond with a symmetry-equivalent hfac fluorine atom (C(13)(–H)···F(20) [2 - *x*,1 - *y*,-*z*] distance of 3.430 (6) Å) (Figure 2). Through the linkage of these intra- and intermolecular C–H···F interactions, a two-dimensional layer is formed in the (010)-plane. These layers are further building up a three-dimensional network, with the hfac CF_3_ groups at the interfaces of the layers, as has been already noticed for other Tb(hfac)_4_ complexes, although with different C_4_mim and C_4_mpyr counterions (Tang & Mudring, 2009) (Figure 3).

### Experimental

General synthetic procedues for the synthesis of rare-earth β-diketonate complexes are given in Melby *et al.*, 1964.

The title compound was synthesized by mixing 3.6 ml of a 1 N sodium hydroxide solution with 9 ml of an ethanol (95%*(v/v)*) solution of hexafluoroacetylacetone (0.505 ml, 3.6 mmol) in a 50 ml Erlenmeyer flask at 60 °C. Subsequently, under stirring, 9 ml of aqueous Tb(NO_3_)_3_.5H_2_O solution (0.3906 g, 0.9 mmol) was added dropwise and finally 1.8 ml of aqueous tetraethylammonium chloride solution (0.0705 g, 0.426 mmol) was added dropwise. The mixture was concentrated by heating until the onset of crystallization. Finally, the solution was filtered and kept overnight to stand at room temperature, to allow the formation of single crystals.

### Refinement

All hydrogen atoms were placed at calculated positions and further refined with isotropic temperature factors fixed at 1.2 times *U*_eq_ of the parent atoms (1.5 times for methyl groups). 1,2 and 1,3 distance restraints to target values, together with restrained *U^ij^* components (for the fluorine atoms) had to be added to model the disorder of the CF_3_ group on one of the hfa ligands [refined occupancy factors were 0.575 (4) and 0.425 (4)].

### Crystal data

**Table:**

(C_8_H_20_N)[Tb(C_5_HF_6_O_2_)_4_]	*F*(000) = 2176
*M**_r_* = 1117.41	*D*_x_ = 1.885 Mg m^−^^3^
Monoclinic, *P*2_1_/*n*	Mo *K*α radiation, λ = 0.71073 Å
Hall symbol: -P 2yn	Cell parameters from 3060 reflections
*a* = 12.7113 (9) Å	θ = 2.4–28.4°
*b* = 16.9355 (13) Å	µ = 1.96 mm^−^^1^
*c* = 18.3540 (11) Å	*T* = 100 K
β = 94.657 (6)°	Needle, colourless
*V* = 3938.1 (5) Å^3^	0.4 × 0.1 × 0.1 mm
*Z* = 4	

### Data collection

**Table:**

Agilent SuperNova Dual Cu at zero Atlas diffractometer	6876 independent reflections
Radiation source: SuperNova (Mo) X-ray Source	4772 reflections with *I* > 2σ(*I*)
mirror	*R*_int_ = 0.064
Detector resolution: 10.35 pixels mm^-1^	θ_max_ = 25.0°, θ_min_ = 2.4°
ω scans	*h* = −10→15
Absorption correction: multi-scan (CrysAlis PRO; Agilent, 2010)	*k* = −20→19
*T*_min_ = 0.531, *T*_max_ = 0.820	*l* = −21→21
14178 measured reflections	

### Refinement

**Table:**

Refinement on *F*^2^	Primary atom site location: structure-invariant direct methods
Least-squares matrix: full	Secondary atom site location: difference Fourier map
*R*[*F*^2^ > 2σ(*F*^2^)] = 0.054	Hydrogen site location: inferred from neighbouring sites
*wR*(*F*^2^) = 0.123	H-atom parameters constrained
*S* = 0.98	*w* = 1/[σ^2^(*F*_o_^2^) + (0.0477*P*)^2^] where *P* = (*F*_o_^2^ + 2*F*_c_^2^)/3
6876 reflections	(Δ/σ)_max_ = 0.001
591 parameters	Δρ_max_ = 1.51 e Å^−^^3^
90 restraints	Δρ_min_ = −1.24 e Å^−^^3^

### Special details

**Table:**

Experimental. CrysAlisPro. Empirical absorption correction using spherical harmonics, implemented in SCALE3 ABSPACK scaling algorithm (Agilent Technologies, 2010)
Geometry. All e.s.d.'s (except the e.s.d. in the dihedral angle between two l.s. planes) are estimated using the full covariance matrix. The cell e.s.d.'s are taken into account individually in the estimation of e.s.d.'s in distances, angles and torsion angles; correlations between e.s.d.'s in cell parameters are only used when they are defined by crystal symmetry. An approximate (isotropic) treatment of cell e.s.d.'s is used for estimating e.s.d.'s involving l.s. planes.
Refinement. Refinement of *F*^2^ against ALL reflections. The weighted *R*-factor *wR* and goodness of fit *S* are based on *F*^2^, conventional *R*-factors *R* are based on *F*, with *F* set to zero for negative *F*^2^. The threshold expression of *F*^2^ > σ(*F*^2^) is used only for calculating *R*-factors(gt) *etc*. and is not relevant to the choice of reflections for refinement. *R*-factors based on *F*^2^ are statistically about twice as large as those based on *F*, and *R*- factors based on ALL data will be even larger.

### Fractional atomic coordinates and isotropic or equivalent isotropic displacement parameters (Å^2^)

**Table:**

	*x*	*y*	*z*	*U*_iso_*/*U*_eq_	Occ. (<1)
C1	0.8770 (4)	−0.0312 (3)	0.0638 (2)	0.0408 (18)	
C2	0.8119 (5)	0.0356 (3)	0.0246 (3)	0.0269 (14)	
C3	0.7175 (5)	0.0124 (3)	−0.0131 (3)	0.0344 (16)	
H3	0.6992	−0.0420	−0.0135	0.041*	
C4	0.6495 (4)	0.0647 (3)	−0.0499 (3)	0.0262 (14)	
C5	0.5548 (5)	0.0315 (4)	−0.0952 (3)	0.0397 (17)	
C6	0.6132 (4)	0.1453 (3)	0.2003 (3)	0.0259 (14)	
C7	0.6239 (5)	0.1927 (3)	0.1298 (2)	0.0251 (14)	
C8	0.5408 (4)	0.2398 (3)	0.1044 (3)	0.0206 (13)	
H8	0.4770	0.2379	0.1280	0.025*	
C9	0.5479 (5)	0.2907 (3)	0.0443 (3)	0.0240 (14)	
C10	0.4618 (5)	0.3541 (3)	0.0308 (3)	0.0299 (15)	
C11	0.8151 (6)	0.4535 (3)	0.1453 (3)	0.0407 (18)	
C12	0.8176 (5)	0.4065 (3)	0.0732 (3)	0.0314 (15)	
C13	0.8257 (5)	0.4492 (3)	0.0096 (3)	0.0414 (18)	
H13	0.8368	0.5046	0.0127	0.050*	
C14	0.8179 (5)	0.4129 (3)	−0.0592 (3)	0.0292 (15)	
C15	0.8325 (5)	0.4656 (3)	−0.1259 (3)	0.0400 (18)	
C16	1.1411 (5)	0.2831 (3)	−0.0144 (3)	0.0295 (15)	
C17	1.0321 (4)	0.2525 (3)	−0.0428 (3)	0.0227 (14)	
C18	1.0187 (4)	0.2291 (3)	−0.1155 (3)	0.0240 (14)	
H18	1.0745	0.2370	−0.1462	0.029*	
C19	0.9261 (4)	0.1948 (3)	−0.1440 (3)	0.0204 (13)	
C20	0.9233 (5)	0.1624 (3)	−0.2223 (3)	0.0301 (15)	
C21	0.0392 (5)	0.1360 (3)	0.3084 (3)	0.0389 (17)	
H21A	0.0257	0.1631	0.3546	0.047*	
H21B	0.1117	0.1143	0.3145	0.047*	
C22	−0.0386 (6)	0.0668 (4)	0.2971 (3)	0.053 (2)	
H22A	−0.1111	0.0870	0.2946	0.080*	
H22B	−0.0276	0.0298	0.3380	0.080*	
H22C	−0.0268	0.0396	0.2513	0.080*	
C23	−0.0751 (5)	0.2336 (4)	0.2359 (3)	0.0406 (17)	
H23A	−0.0736	0.2741	0.1970	0.049*	
H23B	−0.1255	0.1921	0.2179	0.049*	
C24	−0.1160 (6)	0.2718 (4)	0.3030 (4)	0.058 (2)	
H24A	−0.1166	0.2327	0.3423	0.086*	
H24B	−0.1878	0.2914	0.2908	0.086*	
H24C	−0.0700	0.3159	0.3191	0.086*	
C25	0.1164 (5)	0.2586 (3)	0.2736 (3)	0.0367 (17)	
H25A	0.1862	0.2328	0.2823	0.044*	
H25B	0.0976	0.2809	0.3207	0.044*	
C27	0.0568 (5)	0.1617 (4)	0.1757 (3)	0.0431 (18)	
H27A	0.0536	0.2041	0.1385	0.052*	
H27B	0.0007	0.1230	0.1609	0.052*	
C28	0.1652 (5)	0.1201 (4)	0.1762 (3)	0.049 (2)	
H28A	0.2211	0.1569	0.1940	0.074*	
H28B	0.1772	0.1031	0.1265	0.074*	
H28C	0.1662	0.0740	0.2085	0.074*	
C26	0.1247 (5)	0.3262 (3)	0.2183 (3)	0.050 (2)	
H26A	0.1509	0.3053	0.1734	0.076*	
H26B	0.1737	0.3664	0.2393	0.076*	
H26C	0.0550	0.3498	0.2071	0.076*	
N1	0.0347 (3)	0.1967 (2)	0.2478 (2)	0.0238 (11)	
O1	0.8526 (3)	0.1026 (2)	0.03109 (17)	0.0249 (9)	
O2	0.6578 (3)	0.1386 (2)	−0.05394 (18)	0.0276 (10)	
O3	0.7119 (3)	0.1847 (2)	0.10428 (17)	0.0260 (9)	
O4	0.6180 (3)	0.2930 (2)	0.00045 (17)	0.0266 (10)	
O5	0.8142 (3)	0.3344 (2)	0.08163 (17)	0.0271 (10)	
O6	0.7992 (3)	0.34237 (19)	−0.07428 (17)	0.0243 (9)	
O7	0.9674 (3)	0.24911 (18)	0.00547 (17)	0.0221 (9)	
O8	0.8409 (3)	0.18631 (19)	−0.11522 (17)	0.0233 (9)	
F1B	0.8437 (7)	−0.0451 (6)	0.1273 (3)	0.069 (3)	0.425 (4)
F2B	0.9779 (5)	−0.0073 (4)	0.0802 (4)	0.044 (2)	0.425 (4)
F3B	0.8865 (8)	−0.0927 (4)	0.0223 (4)	0.060 (3)	0.425 (4)
F1A	0.8147 (5)	−0.0854 (3)	0.0929 (3)	0.0524 (18)	0.575 (4)
F2A	0.9494 (6)	−0.0096 (4)	0.1126 (3)	0.069 (2)	0.575 (4)
F3A	0.9235 (5)	−0.0736 (3)	0.0135 (3)	0.0427 (18)	0.575 (4)
F4	0.5671 (3)	0.0363 (2)	−0.16613 (18)	0.0701 (13)	
F5	0.5332 (3)	−0.0435 (2)	−0.07899 (19)	0.0610 (12)	
F6	0.4669 (3)	0.0730 (2)	−0.0848 (2)	0.0661 (13)	
F7	0.6745 (3)	0.17776 (19)	0.25596 (15)	0.0388 (9)	
F8	0.6463 (3)	0.07081 (17)	0.19320 (15)	0.0371 (9)	
F9	0.5163 (3)	0.14256 (19)	0.22091 (16)	0.0390 (9)	
F10	0.4251 (3)	0.3581 (2)	−0.03773 (16)	0.0535 (11)	
F11	0.5033 (3)	0.4249 (2)	0.0478 (2)	0.0693 (14)	
F12	0.3802 (3)	0.3450 (2)	0.07027 (17)	0.0517 (11)	
F13	0.8205 (4)	0.5301 (2)	0.13763 (19)	0.0816 (15)	
F14	0.8916 (3)	0.4309 (2)	0.19396 (16)	0.0477 (11)	
F15	0.7254 (3)	0.4379 (3)	0.17600 (19)	0.0700 (13)	
F16	0.9110 (3)	0.5157 (2)	−0.11361 (19)	0.0645 (12)	
F17	0.8480 (4)	0.4257 (2)	−0.18469 (18)	0.0783 (15)	
F18	0.7465 (4)	0.5087 (2)	−0.1421 (2)	0.0771 (14)	
F19	1.1866 (3)	0.2368 (2)	0.03637 (18)	0.0454 (10)	
F20	1.1322 (3)	0.3534 (2)	0.0180 (2)	0.0636 (13)	
F21	1.2065 (3)	0.2928 (3)	−0.06505 (18)	0.0653 (13)	
F22	0.8784 (3)	0.09067 (18)	−0.22706 (16)	0.0432 (10)	
F23	1.0188 (3)	0.1542 (2)	−0.24729 (15)	0.0422 (10)	
F24	0.8672 (3)	0.20959 (19)	−0.26958 (14)	0.0343 (9)	
Tb1	0.78328 (2)	0.228824 (14)	−0.002340 (13)	0.02031 (7)	

### Atomic displacement parameters (Å^2^)

**Table:**

	*U*^11^	*U*^22^	*U*^33^	*U*^12^	*U*^13^	*U*^23^
C1	0.062 (5)	0.020 (3)	0.040 (3)	0.002 (3)	0.000 (3)	−0.007 (3)
C2	0.037 (4)	0.024 (3)	0.021 (3)	0.008 (3)	0.008 (2)	0.003 (2)
C3	0.051 (4)	0.020 (3)	0.029 (3)	0.002 (3)	−0.011 (3)	−0.009 (2)
C4	0.032 (3)	0.015 (3)	0.033 (3)	−0.001 (2)	0.013 (3)	0.003 (2)
C5	0.046 (4)	0.037 (3)	0.035 (3)	−0.020 (3)	−0.010 (3)	0.009 (3)
C6	0.024 (3)	0.026 (3)	0.030 (3)	−0.002 (3)	0.010 (2)	0.003 (2)
C7	0.036 (4)	0.025 (3)	0.014 (2)	−0.007 (3)	0.001 (2)	−0.007 (2)
C8	0.018 (3)	0.022 (3)	0.022 (3)	−0.003 (2)	0.006 (2)	−0.005 (2)
C9	0.035 (3)	0.017 (3)	0.020 (3)	0.002 (2)	0.001 (2)	−0.004 (2)
C10	0.036 (4)	0.029 (3)	0.025 (3)	0.003 (3)	0.003 (3)	−0.002 (3)
C11	0.075 (5)	0.023 (3)	0.023 (3)	0.012 (3)	−0.002 (3)	−0.003 (3)
C12	0.039 (4)	0.028 (3)	0.028 (3)	0.003 (3)	0.004 (3)	−0.004 (3)
C13	0.077 (5)	0.021 (3)	0.027 (3)	0.001 (3)	0.008 (3)	−0.005 (3)
C14	0.042 (4)	0.012 (3)	0.034 (3)	0.005 (3)	0.004 (3)	0.009 (2)
C15	0.052 (4)	0.026 (3)	0.042 (3)	0.001 (3)	0.004 (3)	0.007 (3)
C16	0.028 (3)	0.029 (3)	0.033 (3)	−0.012 (3)	0.007 (3)	0.002 (3)
C17	0.032 (3)	0.012 (2)	0.025 (3)	−0.001 (2)	0.005 (2)	0.001 (2)
C18	0.027 (3)	0.024 (3)	0.021 (3)	−0.005 (3)	0.008 (2)	0.009 (2)
C19	0.030 (3)	0.010 (2)	0.021 (3)	0.001 (2)	0.003 (2)	0.002 (2)
C20	0.038 (4)	0.031 (3)	0.023 (3)	0.002 (3)	0.012 (3)	−0.001 (2)
C21	0.051 (4)	0.027 (3)	0.041 (3)	0.008 (3)	0.015 (3)	0.011 (3)
C22	0.074 (5)	0.030 (3)	0.057 (4)	−0.022 (3)	0.011 (4)	0.005 (3)
C23	0.039 (4)	0.038 (3)	0.045 (3)	−0.003 (3)	0.000 (3)	0.006 (3)
C24	0.059 (5)	0.049 (4)	0.069 (4)	−0.008 (4)	0.032 (4)	−0.022 (4)
C25	0.041 (4)	0.036 (3)	0.033 (3)	−0.007 (3)	0.002 (3)	−0.005 (3)
C27	0.054 (4)	0.041 (4)	0.034 (3)	−0.012 (3)	0.005 (3)	−0.007 (3)
C28	0.066 (5)	0.036 (4)	0.048 (4)	−0.001 (3)	0.022 (3)	−0.013 (3)
C26	0.052 (4)	0.025 (3)	0.077 (4)	−0.004 (3)	0.023 (4)	0.014 (3)
N1	0.027 (3)	0.026 (2)	0.019 (2)	−0.006 (2)	0.0032 (19)	−0.0013 (19)
O1	0.029 (2)	0.0205 (19)	0.0264 (18)	0.0043 (17)	0.0078 (16)	0.0028 (16)
O2	0.026 (2)	0.032 (2)	0.0256 (18)	−0.0020 (18)	0.0034 (16)	0.0074 (17)
O3	0.029 (2)	0.0207 (18)	0.0304 (19)	0.0034 (17)	0.0129 (17)	0.0044 (16)
O4	0.032 (2)	0.0250 (19)	0.0242 (18)	0.0027 (17)	0.0092 (17)	0.0089 (16)
O5	0.034 (2)	0.023 (2)	0.0253 (19)	0.0037 (18)	0.0094 (17)	−0.0015 (16)
O6	0.025 (2)	0.0208 (18)	0.0280 (19)	−0.0021 (17)	0.0061 (16)	0.0069 (16)
O7	0.030 (2)	0.0121 (17)	0.0242 (18)	−0.0036 (16)	0.0042 (17)	−0.0036 (15)
O8	0.028 (2)	0.0177 (18)	0.0242 (18)	−0.0004 (17)	0.0051 (16)	−0.0004 (16)
F1B	0.070 (5)	0.081 (5)	0.056 (4)	0.013 (4)	0.012 (4)	0.028 (4)
F2B	0.055 (5)	0.038 (4)	0.038 (4)	0.014 (4)	−0.009 (4)	0.004 (3)
F3B	0.068 (5)	0.033 (4)	0.077 (5)	0.005 (4)	−0.010 (4)	−0.003 (4)
F1A	0.053 (4)	0.044 (3)	0.062 (3)	0.010 (3)	0.019 (3)	0.037 (3)
F2A	0.094 (5)	0.045 (4)	0.060 (4)	0.008 (4)	−0.046 (4)	0.003 (3)
F3A	0.047 (4)	0.033 (3)	0.051 (3)	0.014 (3)	0.022 (3)	0.008 (3)
F4	0.097 (3)	0.080 (3)	0.0300 (19)	−0.047 (2)	−0.013 (2)	0.0041 (19)
F5	0.084 (3)	0.0315 (19)	0.062 (2)	−0.023 (2)	−0.031 (2)	0.0131 (18)
F6	0.045 (3)	0.066 (3)	0.083 (3)	−0.020 (2)	−0.019 (2)	0.014 (2)
F7	0.046 (2)	0.049 (2)	0.0212 (15)	−0.0158 (18)	0.0030 (15)	0.0005 (15)
F8	0.056 (2)	0.0237 (16)	0.0318 (16)	0.0019 (16)	0.0073 (16)	0.0088 (14)
F9	0.034 (2)	0.047 (2)	0.0382 (17)	−0.0094 (17)	0.0149 (15)	0.0119 (15)
F10	0.047 (2)	0.086 (3)	0.0270 (17)	0.032 (2)	0.0002 (16)	0.0033 (18)
F11	0.054 (3)	0.0249 (19)	0.126 (3)	0.0111 (19)	−0.011 (2)	−0.012 (2)
F12	0.046 (2)	0.069 (3)	0.0436 (18)	0.0258 (19)	0.0239 (17)	0.0135 (18)
F13	0.172 (4)	0.030 (2)	0.040 (2)	0.017 (2)	−0.006 (2)	−0.0085 (17)
F14	0.064 (3)	0.045 (2)	0.0326 (18)	0.0107 (19)	−0.0061 (18)	−0.0105 (16)
F15	0.056 (3)	0.105 (3)	0.050 (2)	0.018 (2)	0.0146 (19)	−0.040 (2)
F16	0.081 (3)	0.058 (2)	0.053 (2)	−0.036 (2)	0.004 (2)	0.021 (2)
F17	0.171 (4)	0.035 (2)	0.0339 (19)	−0.021 (3)	0.036 (2)	0.0034 (17)
F18	0.085 (3)	0.070 (3)	0.077 (3)	0.018 (3)	0.013 (2)	0.051 (2)
F19	0.041 (2)	0.047 (2)	0.0453 (19)	−0.0115 (18)	−0.0128 (17)	0.0125 (17)
F20	0.053 (3)	0.033 (2)	0.102 (3)	−0.0132 (19)	−0.005 (2)	−0.020 (2)
F21	0.036 (2)	0.124 (4)	0.0367 (19)	−0.038 (2)	0.0125 (17)	0.001 (2)
F22	0.067 (3)	0.0304 (18)	0.0317 (17)	−0.0021 (18)	0.0037 (17)	−0.0135 (15)
F23	0.041 (2)	0.059 (2)	0.0284 (17)	0.0141 (18)	0.0105 (15)	−0.0070 (16)
F24	0.036 (2)	0.049 (2)	0.0180 (15)	0.0086 (16)	0.0026 (14)	0.0032 (15)
Tb1	0.02640 (15)	0.01570 (12)	0.01963 (12)	0.00014 (12)	0.00668 (10)	0.00082 (11)

### Geometric parameters (Å, °)

**Table:**

C1—F2A	1.284 (6)	C17—O7	1.258 (6)
C1—F1B	1.293 (7)	C17—C18	1.390 (7)
C1—F3B	1.300 (7)	C18—C19	1.376 (7)
C1—F3A	1.344 (6)	C18—H18	0.9500
C1—F1A	1.350 (6)	C19—O8	1.251 (6)
C1—F2B	1.356 (7)	C19—C20	1.538 (7)
C1—C2	1.545 (7)	C20—F23	1.339 (6)
C2—O1	1.249 (6)	C20—F22	1.341 (6)
C2—C3	1.393 (8)	C20—F24	1.341 (6)
C3—C4	1.375 (8)	C21—N1	1.513 (7)
C3—H3	0.9500	C21—C22	1.536 (8)
C4—O2	1.259 (6)	C21—H21A	0.9900
C4—C5	1.514 (8)	C21—H21B	0.9900
C5—F4	1.326 (6)	C22—H22A	0.9800
C5—F5	1.338 (6)	C22—H22B	0.9800
C5—F6	1.348 (7)	C22—H22C	0.9800
C6—F9	1.318 (6)	C23—C24	1.520 (8)
C6—F8	1.340 (6)	C23—N1	1.528 (7)
C6—F7	1.350 (6)	C23—H23A	0.9900
C6—C7	1.537 (7)	C23—H23B	0.9900
C7—O3	1.255 (6)	C24—H24A	0.9800
C7—C8	1.375 (7)	C24—H24B	0.9800
C8—C9	1.410 (7)	C24—H24C	0.9800
C8—H8	0.9500	C25—N1	1.524 (7)
C9—O4	1.249 (6)	C25—C26	1.539 (8)
C9—C10	1.539 (8)	C25—H25A	0.9900
C10—F10	1.307 (6)	C25—H25B	0.9900
C10—F12	1.322 (6)	C27—N1	1.498 (7)
C10—F11	1.336 (6)	C27—C28	1.547 (9)
C11—F13	1.307 (6)	C27—H27A	0.9900
C11—F14	1.322 (7)	C27—H27B	0.9900
C11—F15	1.337 (8)	C28—H28A	0.9800
C11—C12	1.547 (7)	C28—H28B	0.9800
C12—O5	1.233 (6)	C28—H28C	0.9800
C12—C13	1.384 (7)	C26—H26A	0.9800
C13—C14	1.401 (7)	C26—H26B	0.9800
C13—H13	0.9500	C26—H26C	0.9800
C14—O6	1.244 (6)	Tb1—O1	2.373 (3)
C14—C15	1.538 (8)	Tb1—O2	2.351 (4)
C15—F17	1.302 (7)	Tb1—O3	2.345 (3)
C15—F16	1.315 (7)	Tb1—O4	2.369 (4)
C15—F18	1.328 (7)	Tb1—O5	2.372 (3)
C16—F21	1.307 (6)	Tb1—O6	2.351 (3)
C16—F19	1.315 (6)	Tb1—O7	2.359 (4)
C16—F20	1.340 (6)	Tb1—O8	2.365 (3)
C16—C17	1.531 (8)		
			
F2A—C1—F1B	72.1 (5)	F23—C20—C19	113.8 (5)
F2A—C1—F3B	122.7 (6)	F22—C20—C19	111.2 (4)
F1B—C1—F3B	115.8 (6)	F24—C20—C19	111.3 (4)
F2A—C1—F3A	107.5 (5)	N1—C21—C22	115.7 (5)
F1B—C1—F3A	135.8 (6)	N1—C21—H21A	108.4
F2A—C1—F1A	109.2 (5)	C22—C21—H21A	108.4
F3B—C1—F1A	76.9 (5)	N1—C21—H21B	108.4
F3A—C1—F1A	102.4 (5)	C22—C21—H21B	108.4
F1B—C1—F2B	103.2 (6)	H21A—C21—H21B	107.4
F3B—C1—F2B	103.7 (6)	C21—C22—H22A	109.5
F3A—C1—F2B	81.4 (5)	C21—C22—H22B	109.5
F1A—C1—F2B	133.7 (5)	H22A—C22—H22B	109.5
F2A—C1—C2	116.2 (5)	C21—C22—H22C	109.5
F1B—C1—C2	110.5 (5)	H22A—C22—H22C	109.5
F3B—C1—C2	112.7 (5)	H22B—C22—H22C	109.5
F3A—C1—C2	108.6 (4)	C24—C23—N1	115.2 (5)
F1A—C1—C2	111.9 (4)	C24—C23—H23A	108.5
F2B—C1—C2	110.2 (5)	N1—C23—H23A	108.5
O1—C2—C3	129.5 (5)	C24—C23—H23B	108.5
O1—C2—C1	114.9 (5)	N1—C23—H23B	108.5
C3—C2—C1	115.6 (5)	H23A—C23—H23B	107.5
C4—C3—C2	123.0 (5)	C23—C24—H24A	109.5
C4—C3—H3	118.5	C23—C24—H24B	109.5
C2—C3—H3	118.5	H24A—C24—H24B	109.5
O2—C4—C3	128.0 (5)	C23—C24—H24C	109.5
O2—C4—C5	113.8 (5)	H24A—C24—H24C	109.5
C3—C4—C5	118.1 (5)	H24B—C24—H24C	109.5
F4—C5—F5	108.6 (5)	N1—C25—C26	112.8 (5)
F4—C5—F6	105.9 (5)	N1—C25—H25A	109.0
F5—C5—F6	106.0 (5)	C26—C25—H25A	109.0
F4—C5—C4	111.4 (5)	N1—C25—H25B	109.0
F5—C5—C4	113.5 (5)	C26—C25—H25B	109.0
F6—C5—C4	111.1 (5)	H25A—C25—H25B	107.8
F9—C6—F8	107.6 (4)	N1—C27—C28	113.9 (5)
F9—C6—F7	107.0 (4)	N1—C27—H27A	108.8
F8—C6—F7	106.8 (4)	C28—C27—H27A	108.8
F9—C6—C7	114.1 (4)	N1—C27—H27B	108.8
F8—C6—C7	111.1 (4)	C28—C27—H27B	108.8
F7—C6—C7	109.8 (4)	H27A—C27—H27B	107.7
O3—C7—C8	128.3 (5)	C27—C28—H28A	109.5
O3—C7—C6	113.7 (5)	C27—C28—H28B	109.5
C8—C7—C6	118.0 (5)	H28A—C28—H28B	109.5
C7—C8—C9	121.7 (5)	C27—C28—H28C	109.5
C7—C8—H8	119.2	H28A—C28—H28C	109.5
C9—C8—H8	119.2	H28B—C28—H28C	109.5
O4—C9—C8	128.1 (5)	C25—C26—H26A	109.5
O4—C9—C10	114.2 (4)	C25—C26—H26B	109.5
C8—C9—C10	117.6 (5)	H26A—C26—H26B	109.5
F10—C10—F12	107.4 (5)	C25—C26—H26C	109.5
F10—C10—F11	106.4 (5)	H26A—C26—H26C	109.5
F12—C10—F11	106.8 (4)	H26B—C26—H26C	109.5
F10—C10—C9	112.6 (4)	C27—N1—C21	112.4 (4)
F12—C10—C9	114.2 (4)	C27—N1—C25	112.0 (4)
F11—C10—C9	109.1 (5)	C21—N1—C25	104.8 (4)
F13—C11—F14	108.5 (5)	C27—N1—C23	105.6 (4)
F13—C11—F15	107.2 (5)	C21—N1—C23	111.3 (4)
F14—C11—F15	105.3 (4)	C25—N1—C23	110.8 (4)
F13—C11—C12	114.4 (5)	C2—O1—Tb1	130.7 (3)
F14—C11—C12	111.3 (5)	C4—O2—Tb1	132.8 (3)
F15—C11—C12	109.7 (5)	C7—O3—Tb1	134.0 (3)
O5—C12—C13	129.0 (5)	C9—O4—Tb1	132.9 (3)
O5—C12—C11	113.5 (4)	C12—O5—Tb1	132.2 (3)
C13—C12—C11	117.5 (5)	C14—O6—Tb1	133.1 (3)
C12—C13—C14	121.8 (5)	C17—O7—Tb1	131.8 (3)
C12—C13—H13	119.1	C19—O8—Tb1	132.4 (3)
C14—C13—H13	119.1	O3—Tb1—O6	141.84 (12)
O6—C14—C13	128.3 (5)	O3—Tb1—O2	80.42 (12)
O6—C14—C15	114.4 (5)	O6—Tb1—O2	113.06 (12)
C13—C14—C15	117.2 (5)	O3—Tb1—O7	116.42 (12)
F17—C15—F16	108.3 (5)	O6—Tb1—O7	77.57 (11)
F17—C15—F18	106.0 (5)	O2—Tb1—O7	139.15 (12)
F16—C15—F18	106.4 (5)	O3—Tb1—O8	143.35 (12)
F17—C15—C14	113.2 (5)	O6—Tb1—O8	73.15 (11)
F16—C15—C14	112.2 (5)	O2—Tb1—O8	72.40 (12)
F18—C15—C14	110.3 (5)	O7—Tb1—O8	73.80 (12)
F21—C16—F19	108.1 (5)	O3—Tb1—O4	74.01 (12)
F21—C16—F20	106.6 (5)	O6—Tb1—O4	75.75 (12)
F19—C16—F20	105.3 (4)	O2—Tb1—O4	74.53 (12)
F21—C16—C17	114.3 (4)	O7—Tb1—O4	144.01 (11)
F19—C16—C17	111.9 (4)	O8—Tb1—O4	119.80 (12)
F20—C16—C17	110.1 (5)	O3—Tb1—O5	75.68 (12)
O7—C17—C18	128.9 (5)	O6—Tb1—O5	74.41 (11)
O7—C17—C16	113.6 (4)	O2—Tb1—O5	145.33 (12)
C18—C17—C16	117.4 (5)	O7—Tb1—O5	74.91 (12)
C19—C18—C17	121.3 (5)	O8—Tb1—O5	138.75 (12)
C19—C18—H18	119.4	O4—Tb1—O5	74.97 (12)
C17—C18—H18	119.4	O3—Tb1—O1	69.99 (11)
O8—C19—C18	129.2 (5)	O6—Tb1—O1	146.60 (12)
O8—C19—C20	113.5 (4)	O2—Tb1—O1	75.19 (12)
C18—C19—C20	117.4 (5)	O7—Tb1—O1	76.69 (12)
F23—C20—F22	106.1 (4)	O8—Tb1—O1	79.48 (11)
F23—C20—F24	106.8 (4)	O4—Tb1—O1	135.94 (12)
F22—C20—F24	107.3 (4)	O5—Tb1—O1	118.18 (11)

### Hydrogen-bond geometry (Å, °)

**Table:**

*D*—H···*A*	*D*—H	H···*A*	*D*···*A*	*D*—H···*A*
C3—H3···F5	0.95	2.34	2.718 (7)	103
C8—H8···F9	0.95	2.37	2.737 (6)	102
C8—H8···F12	0.95	2.39	2.743 (6)	102
C13—H13···F13	0.95	2.36	2.726 (6)	102
C13—H13···F20^i^	0.95	2.51	3.430 (6)	164
C18—H18···F21	0.95	2.35	2.713 (7)	102
C18—H18···F23	0.95	2.39	2.731 (6)	101
C21—H21A···F10^ii^	0.99	2.47	3.279 (7)	139
C26—H26C···F14^iii^	0.98	2.49	3.451 (7)	169
C27—H27B···F2A^iii^	0.99	2.48	3.371 (9)	149

Symmetry codes: (i) −*x*+2, −*y*+1, −*z*; (ii) *x*−1/2, −*y*+1/2, *z*+1/2; (iii) *x*−1, *y*, *z*.
